# *DEEP-SEE*: Joint Object Detection, Tracking and Recognition with Application to Visually Impaired Navigational Assistance

**DOI:** 10.3390/s17112473

**Published:** 2017-10-28

**Authors:** Ruxandra Tapu, Bogdan Mocanu, Titus Zaharia

**Affiliations:** 1 Advanced Research and TEchniques for Multidimensional Imaging Systems Department, Institut Mines-Télécom/Télécom SudParis, UMR CNRS MAP5 8145 and 5157 SAMOVAR, 9 rue Charles Fourier, 91000 Évry, France; bogdan.mocanu@telecom-sudparis.eu or bcmocanu@comm.pub.ro (B.M.); titus.zaharia@telecom-sudparis.eu (T.Z.); 2 Telecommunication Department, Faculty of ETTI, University “Politehnica” of Bucharest, SplaiulIndependentei 313, 060042 Bucharest, Romania

**Keywords:** object detection, tracking and recognition, convolutional neural networks, visually impaired users, wearable assistive device

## Abstract

In this paper, we introduce the so-called ***DEEP-SEE*** framework that jointly exploits computer vision algorithms and deep convolutional neural networks (CNNs) to detect, track and recognize in real time objects encountered during navigation in the outdoor environment. A first feature concerns an object detection technique designed to localize both static and dynamic objects without any a priori knowledge about their position, type or shape. The methodological core of the proposed approach relies on a novel object tracking method based on two convolutional neural networks trained offline. The key principle consists of alternating between tracking using motion information and predicting the object location in time based on visual similarity. The validation of the tracking technique is performed on standard benchmark VOT datasets, and shows that the proposed approach returns state-of-the-art results while minimizing the computational complexity. Then, the ***DEEP-SEE*** framework is integrated into a novel assistive device, designed to improve cognition of VI people and to increase their safety when navigating in crowded urban scenes. The validation of our assistive device is performed on a video dataset with 30 elements acquired with the help of VI users. The proposed system shows high accuracy (>90%) and robustness (>90%) scores regardless on the scene dynamics.

## 1. Introduction

Although object tracking represents a fundamental problem in the computer vision community due to the wide range of related applications (e.g., automatic video surveillance, wearable assistive devices, robots navigation, structure from motion or human-machine interaction), it is still an open issue of research. Despite significant progress achieved in the last decade, tremendous challenges still exists in designing a robust object tracker able to handle important changes in viewpoint, object motion, light variation, pose variation, object occlusion or background clutter.

In this paper, we propose a novel joint object detection/tracking/recognition framework, called ***DEEP-SEE***, based on computer vision algorithms and offline trained deep convolutional neural networks. The proposed framework is integrated within an assistive device, dedicated to visually impaired (VI) users. The major contributions of the paper are summarized below.

A first feature concern the detection and recognition module, which is based on the YOLO (You Only Look Once) [1] algorithm. In its original form, the YOLO algorithm is designed to deal solely with spatial objects, without taking into account the spatio-temporal evolution of the detected objects. In addition, it cannot guarantee that the same object is detected in each frame of a video sequence. Moreover, the category detected for a given object can vary over time (due to miss classification) and the recognition becomes unreliable. To overcome such limitations, a first contribution is related to the combination of YOLO with an object tracking procedure. The tracking procedure makes it possible to fulfill the missing information in the case where YOLO is failing but also to analyze the detected categories over time and to identify reliably the dominant category labels.

The second contribution, which represents the methodological core of the paper, concerns the object tracking approach. The proposed method uses two convolutional neural networks (CNNs) trained offline with both motion and visual patterns. We show how an adaptive visual appearance model can be constructed on-the-fly, without the need of any on-line learning procedures. In addition, we introduce an occlusion detection and handling strategy. In this way, the method is able to handle important or complete object occlusions, object movement or camera drift.

The third contribution is the integration of the object detection/tracking/recognition framework in a novel VI-dedicated assistive device, denoted by ***DEEP-SEE*** navigational assistant. The YOLO algorithm has been here extended and trained with novel categories specific for an assistive device dedicated to the VI. We make sure that the system can localize, in real-time, both static obstructions (e.g., pillars, fences, traffic signs, stairs, trees, benches, etc.) and dynamic objects (e.g., vehicles, bicycles, motorcycles and pedestrians). The proposed system is able to acquire information from the environment, process, interpret it and transmit alert messages to the VI user in order to avoid dangerous situations or possible collisions. Let us underline the real-time constraints that are necessary to consider and achieve within the framework of this VI-related application which strongly condition the whole processing chain and impact the technical choices retained.

The rest of the paper is organized as follows. In Section 2, we review the state-of-the-art object detection and tracking methods based on computer vision/machine learning algorithms. In Section 3, we introduce the proposed architecture, denoted by ***DEEP-SEE***, and we describe the main steps involved: obstacle detection, tracking and recognition. Section 4 presents the experimental evaluation of the tracking system performed on standard benchmarks (VOT2016). In Section 5, we describe the ***DEEP-SEE*** navigational assistant, with the proposed hardware/software architecture, main features and performance evaluation. We show that it is possible to obtain high accuracy and recall rates with a computationally efficient approach that notably achieves real-time performances on wearable devices. Finally, Section 6 concludes the paper and opens novel directions for further work and developments.

## 2. Related Work

Since more than two decades ago, numerous object detection and tracking algorithms have been proposed. In this section, we focus our attention solely on recent discriminative algorithms that have been introduced in the last couple of years. Modern approaches [2] dedicated to adaptive object tracking techniques typically use classifiers to differentiate targets from the surrounding background information. Various methods [3,4,5,6,7] update the object appearance model by taking into account the dynamic scene changes. Even though the CNN networks are highly effective in object detection tasks, within the context of target tracking, the CNN proves to be difficult to train because of noisy labeled data that can lead to overfitting problems, notably when the number of training examples is too small.

In [2], the scale-adaptive mean-shift (ASMS) tracker based on the Heinger distance is introduced. The system shows high robustness to relatively important scale changes such as: scale expansions due to background clutter or scale implosion due to self-similar objects situated in the neighboring area.

In [3], an object tracking system that combines several independent and heterogeneous tracking methods is proposed. The system identifies an outlier subset of positions, based on the “Median Absolute Deviations” (MAD) measure, in order to determine the optimal location of the object. The MAD fusion strategy is very generic and only requires frame-based object bounding boxes as input.

The TricTRACK [4] system uses a part-based tracking method in order to replace the local matching of an appearance model by the direct prediction of the object displacement. TricTRACK uses a regression model with incremental learning to track arbitrary shape objects.

In [5], the KCF2014 tracker is introduced. An analytic model trained on a datasets of thousands of translated and scaled patches is here used in order to cope with natural image changes. The system is designed to analyze the video stream in the Fourier domain in order to reduce both the storage and the computational resources. The sequentially training of convolutional networks (SCT4) for object tracking is introduced in [6]. The SCT4 considers the online CNN training as an ensemble and each channel of the output feature map as an individual base learner. Then, each network is trained using a different loss criterion in order to reduce correlation and avoid overfitting. At the end, in order to make a decision all base learners are sequentially sampled into the ensemble. In [7], the SWCF object tracker is introduced. The system uses a method to estimate a spatial window for the object observation, rather the entire frame, in order to reduce the tracker drift. An arbitrary object tracker using adaptive clustered decision trees and dynamic appearance models (CDTT) is introduced in [8]. Targets are analyzed at three levels of granularity: pixel level, part-based level and bounding box level. Then, based on an adaptive clustering decision tree, the system dynamically selects the features to robustly localize the target.

Differently, a color-based object tracker denoted DAT is introduced in [9]. The system uses an object model and a background model to identify potentially distracting regions in advance and to remove or suppress distracters. In [10], a tracker that exploits the dense spatio-temporal context (STC) is introduced. Within the framework of a Bayesian framework, the system models the statistical correlation between the target and surrounding regions.

The tree-structured CNN (TCNN) tracker recently proposed in [11] uses multiple CNNs to estimate the target states and determine the optimal displacement path. The multiple CNNs stored in diverse brunches of the tree structure can treat in a multi-modal manner the various changes in object appearance and reliably preserve the model through smooth updates. Due to its high performances, the TCNN approach is considered today as state of the art in the field [12].

The Staple single object tracker introduced in [13] is based on correlation filters in order to deal with motion blur, illumination changes, color variations and various shape deformation. At the end, the complementary cues considered are combined in a rigid regression framework.

Finally, let us mention the winner of the international VOT 2016 tracking contest [12], which is denoted by C-COT [14]. The C-COT CNN tracker learns discriminative convolution operators in the continuous spatial domain. The system allows the integration of multi-resolution feature maps and enables sub-pixel object localization.

However, the neural networks are complex to train, require important processing resources and are very slow in the test phase. Thus, in terms of speed, the above-mentioned trackers range from 0.8 fps to 10 fps, while the top performing algorithm in the evaluation tests runs at 1 fps on GPU. Hence, such trackers are penalized when considering practical applications. To deal with this drawback, in [15], the GOTURN algorithm is proposed. GOTURN trains offline a CNN with various object motion patterns. In the test phase, the tracker uses a simple feed-forward network and no online training is required. The system allows single object tracking at 50 fps.

In this paper, we introduce an integrated single object detection, recognition and tracking approach that integrates both visual and motion cues to perform accurate object position estimation. The key principle consists of alternating between tracking using motion information and adjusting the predicted location based on visual similarity.

## 3. *DEEP-SEE*: Joint Object Detection, Recognition and Tracking

Similar to any tracking procedure, we first need an object detection approach that can provide an initialization of the tracking process. In our work, we have adopted the YOLO approach, described in the following section.

### 3.1. Object Detection and Recognition

We have adopted an initial object detection and recognition procedure based on the YOLO [1] algorithm. YOLO is a CNN-based approach which is repurposing classifiers to the object detection task. The object detection issue is treated as a regression mechanism for spatially separated bounding boxes and their associated class probabilities. The CNN architecture is inspired by the GoogLeNet model with 24 convolutional layers, followed by two fully connected layers. A final convolutional layer of the network can predict simultaneously multiple bounding boxes and the associated class probabilities. In this way, YOLO jointly performs the object detection and recognition tasks.

The YOLO framework encodes during training and testing the contextual information about the object class and its appearance. Due to the global object representations, the system is less likely to return false alarms or missed detections when applied to novel/unexpected video instances.

To fit the requirements of the considered VI-related application, we have extended YOLO with additional categories, specific to assistive devices for visually impaired users. The system was trained on the standard ImageNet dataset from which we retained as relevant the following object classes: vehicles; bicycles, motorcycles and pedestrians. In addition, we have constructed a set of categories relevant from the perspective of a VI people, including garbage cans, fences, pylons, edge of pavements and descending stairs. These elements have been included in a single, global category called static obstacles. The class of static obstacles can be extended with additional elements (e.g., bushes, tress, branches, etc.), depending on the user needs. However, the ***DEEP-SEE*** system cannot identify descending stairs or holes in the ground, which are difficult to distinguish when using monoscopic vision. To this purpose, the utilization of depth cameras would be more appropriate, but this issue will not be addressed in the paper. The CNN has been trained by using the same training dataset as the one presented in [16], which includes a total number of 4500 images.

To train the network we employed the parameters recommended by authors in [1] that returned the top-1 performances in the evaluation tests. Thus, we used the stochastic gradient descent with a starting learning rate of 0.1 and polynomial rate decay with a power of 4.

YOLO presents the following advantages: (1) the processing time is reduced, the system being able to operate at more than 100 fps when running on an Nvidia 1050Ti GPU; (2) it takes as input the entire image during both training and testing phases and so it encodes contextual information about classes and their appearance; and (3) it has the highest score of correctly detected objects and the lowest number of false positives when compared to state of the art methods [1]. In addition, the detector can be used to predict candidate location for novel objects in video frames.

However, the YOLO algorithm operates exclusively on a spatial basis, and is completely agnostic to the spatio-temporal evolution and thus to the motion/deformation/occlusion that a given object may undergo. Moreover, it cannot be reliably used in all video frames because the objects can exhibit important changes in size/appearance, can be partial or completely occluded or the video stream can become cluttered or noisy due to the users’ own displacement.

To overcome such limitations, we propose an object tracking procedure, which is the key ingredient of the proposed ***DEEP-SEE*** approach. On the one hand, all new objects detected with YOLO serve as initialization to the tracking procedure, and are thus followed in the subsequent video frames. This makes it possible to acquire a continuous and temporally-consistent knowledge about the object’s position and shape. On the other hand, the associated categories recognized by YOLO are analyzed and cross-validated in time, which permits to enhance the overall robustness of the object recognition process.

The tracking procedure proposed is detailed in the following section.

### 3.2. Object Tracking

**Figure 1:**
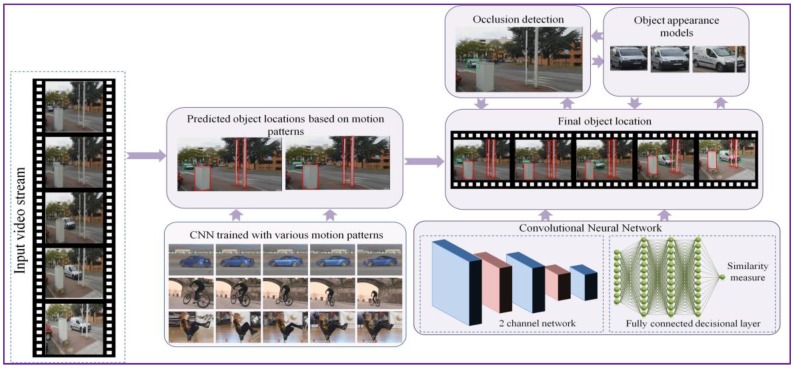
The ***DEEP-SEE*** object tracking framework.

Figure 1 illustrates the object tracking procedure proposed, with the main steps involved: offline training, object tracking using motion patterns and object bounding box adjustment based on visual similarity constraints (that involve occlusion detection and object appearance model). The key principle of the proposed tracking approach consists in alternating between tracking using motion information and predicting the object location in time based on visual similarity. To this purpose, we propose the on-the-fly construction of a visual appearance model that can continuously adapt to the geometric and photometric changes of the object while correctly dealing with the potential occlusions that may occur during its lifetime.

The initial object position estimation is obtained with the help of a CNN trained with motion patterns.

#### 3.2.1. Initial Object Position Estimation: CNN Trained with Motion Patterns

The tracking system takes as input, in a given reference frame, the object’s bounding box predicted by the detection and recognition module (cf. Section 3.1). Then, the objective is to estimate the object’s position in the successive frame.

To obtain an initial estimation of the object’s position, we have adopted the GOTURN [15] network architecture that exploits a regression technique to train offline a CNN with generic relationships between the objects appearances and their associated motion patterns.

**Figure 2:**
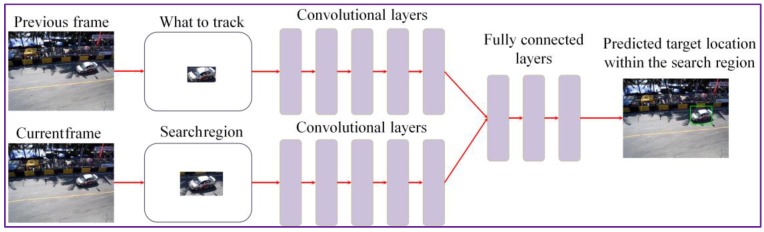
The ***DEEP-SEE*** CNN (convolutional neural network) network architecture used for object tracking.

We performed the offline training of the network on a collection of 312 videos selected from the ALOV 300+ benchmark [17]. Let us underline that none of the videos used for our experimental evaluation (cf. Section 4) have been used in the training process. Here, a subset of frames in each image sequence is labeled with the location of some object. We apply as input to the CNN both the target object (from the previous frame) and the search region from the current frame, centered in the same position as the target. The search region has twice the size of reference object bounding box (Figure 2). The output of the convolution layers is a set of features that capture the image high-level representation. These features are further applied as input to the fully connected layer. By comparing the features associated to the object with those extracted from the search area, we can determine the novel position of the target (Figure 2).

We adopted the CNN architecture of the first five layers of the traditional CaffeNet framework [18]. The layers are pre-trained using the ImageNet database [19]. In the training phase, we adopted a learning rate of 1 × 10^−5^ and we constrained all other parameters to the default values of CaffeNet. The outputs from all the convolutional layers are concatenated in one vector that is applied to the fully connected layers. The CNN has four outputs, which represent the *x,y* coordinates corresponding to the object bounding box upper left and lower right corners. Let us emphasize that the CNN training is performed uniquely in the offline stage. In the online phase, the network weights are frozen and no fine-tuning is required.

Because the proposed tracker learns generic relationships between the object’s appearance model and the motion patterns, various objects can be tracked without requiring any category-specific information about the target. The method proves to be robust when handling objects that undergo various transformations, such as deformation, translation, rotation, occlusion or light changes, and is very fast (more than 50 fps when running on Nvidia 1050Ti GPU). However, the system fails to track fast moving targets that undergo partial or complete occlusion. In addition, the approach fails to deal with multiple moving objects characterized by similar motion patterns that are situated in the same neighborhood. This phenomenon is known as tracker drift [14]. To overcome such limitations, we develop a model that integrates rich visual cues. The object’s bounding box size, position and shape are continuously updated, which makes it possible to prevent the tracker drift, notably in the case of cluttered scenes. The proposed strategy includes two major phases: (1) occlusion detection/handling; and (2) construction of an object appearance model. Let us first describe how the occlusion detection and handling is dealt with.

#### 3.2.2. Occlusion Detection and Handling

**Figure 3:**
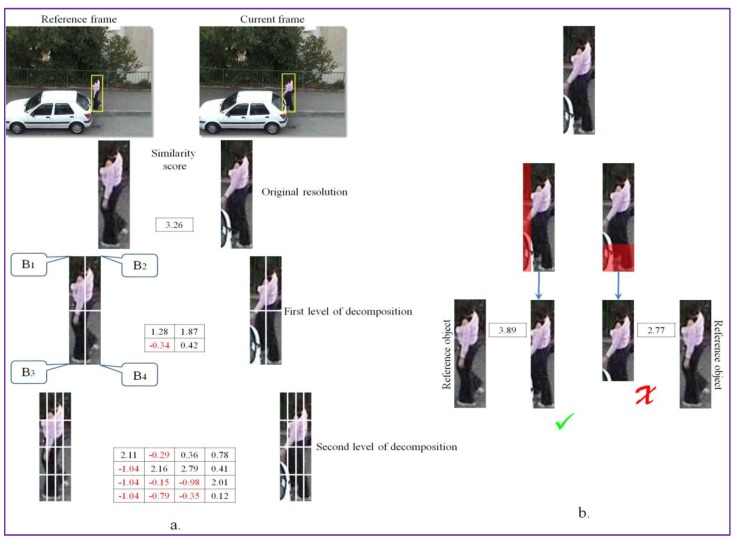
Occlusion detection and handling: (**a**) quad-tree decomposition and comparison using the offline trained CNN; and (**b**) object bounding box adjustment based on maximum similarity score.

The process of occlusion detection and handling is illustrated in Figure 3. The procedure takes as input both the reference object (from the previous frame) and the candidate (in the current frame) bounding box, determined as described in the previous section. Both of them are recursively divided into a set of non-overlapping image patches with the help of a quad-tree decomposition algorithm. The process is recursively repeated until the second level of decomposition. We have chosen to use only two levels of decomposition in order to ensure a “reasonable” degree of descriptiveness for the similarity measure used consequently, which requires the availability of a sufficient number of pixels per image patch.

In the quad-tree subdivision scheme, an extra degree of freedom is introduced, which aims at optimizing the image patch position in each quadrant. Note that the object bounding box (*B*) is a 4 × 4 cell grid that can be interpreted as a set of four quadrants (each quadrant being composed of 2 × 2 cells), denoted by *B_i_* with *i* ∈ {1, 2, 3, 4}. Thus, we can consider that the object’s bonding box is defined as the concatenation of the four quadrants: $B=\left[B_{1},B_{2},B_{3},B_{4}\right]$.

Let us denote by *B* and *B’* the initial image patches in the source and target image, respectively. The patch *B’* is the image patch obtained after applying of the motion-based tracking procedure described in Section 3.2.1.

To cancel the effect of non-rigid or articulated object motion we propose to optimize independently the position of the four quadrants of the target patch *B’* so that to maximize the following similarity score:

(1) $$SimScore\left(B,\;B^{\prime }\right)=\frac{1}{4}\overset{4}{\underset{i=1}{\sum }}\underset{k}{\max}SimScore\left(B_{i},B_{i}^{\prime }\left(v_{k}\right)\right),$$

where ${\left\{v_{k}\right\}}_{k}$ denotes the set of all possible patch displacement vectors within the considered search area and $B_{i}^{\prime }\left(v_{k}\right)$ represents the *i*th quadrant in the target image, displaced by offset $v_{k}$ and SimScore(·) is the similarity metric between the considered image patches.

The search area for all the quadrants is defined as twice the size of the quadrant in both horizontal and vertical directions.

This comparison strategy is recursively applied to each sub-quadrant in order to obtain a finer estimation of the object’s position. Let us note that the recursivity principle considered makes it possible to efficiently compute the corresponding similarity metric. Let us also underline that, by letting the four quadrants move independently, it becomes possible to handle articulated objects with various sub-parts moving in different directions.

The similarity score between the analyzed image patches is computed using the DeepCompare [20] algorithm, a CNN-based method trained to take into account various types of transformations and effects in the image representation (e.g., illumination or wide baseline). The system does not require any manually tuned features and is able to generate a patch similarity function directly from annotated pairs of raw image patches. In our case, we have used the already trained network provided by the authors. We have adopted the two-channel CNN model architecture described in [20], due to its high flexibility, accuracy and processing speed. Here, the two patches under analysis are considered as a two-channel image that is applied directly as input to the first layer of the network. In the bottom part, the CNN architecture is composed of a series of convolutional, ReLU and max-pooling layers. The top module consists of a fully connected layer with a unique output that yields the similarity score between the analyzed image patches. The similarity scores returned by the DeepCompare [20] algorithm vary between [−1.1; +10], where −1.1 signifies that the patches being analyzed show no resemblance, while a +10 value is assigned to identical image patches.

The similarity scores of the adjusted object bounding box are further analyzed to determine the beginning of an occlusion. The occlusion detection is performed on a per-quadrant basis. More precisely, we decide that an occlusion is occurring if at least one of the four quadrants (1st level of decomposition) and of *all* its sub-quadrants (2nd level of decomposition) present negative similarity values. This process is illustrated in Figure 3a. Here, the lower left quadrant and the set of all of its sub-quadrants are providing negative similarity values, which is due to an occlusion.

When an occlusion is detected, we consider that the object bounding box contains some undesired background information or that the target entered in an occluded state. If the size of the object’s bounding box is not adjusted appropriately, such parasite information will bias the tracking process and the object can be completely lost within a few frames.

To overcome this limitation, we propose to trim the object’s bounding box whenever such phenomenon is identified. We consider four trimming operations in the following directions: left (L), right (R), down (D) and up (U). The trimming operation consists in reducing the size of the object bounding box with 1/8 of its original dimension by cutting along one of the considered directions. We adopted a reduction with 1/8 of the original size in order to avoid a too brutal shrinking. If one trimming operation is not sufficient to eliminate all the residual information, the process can be repeated recursively. The algorithm stops when the similarity score, with respect to the reference image patch at the original resolution, stops increasing.

The cutting direction is determined based on the similarity scores of the image patches from the quad-tree decomposition. For the example presented in Figure 3, because negative similarity scores are obtained in the $B_{3}$ region and all the associated sub-patches return negative scores, two trimming operations are evaluated: right and down (Figure 3b). If we denote with $SimScore_{CUT1}$ and $SimScore_{CUT2}$ the visual similarity scores obtained after performing the cut in the considered directions, then we select as the optimal adjustment operation the one that maximizes the following score:

(2) $$MSimScore=\max\left\{SimScore_{CUT1};SimScore_{CUT2}\right\};\;$$

Finally, to validate the adjusted object bounding box, we impose that the MSimScore obtained after the adjustment operation to be superior to the original similarity score computed initially between the image patches at the original resolution. In addition, for objects with bounding boxes inferior to 10 × 10 pixels no cutting operations are allowed, since the similarity measure becomes poorly relevant in this case.

This procedure makes it possible to obtain a refined bounding box that is able to take into account the partial occlusions that can occur, by slightly adjusting the shape of the tracked object. The position of this refined bounding box is still strongly determined by the motion pattern-based approach described in Section 3.2.1. In the case of hard or complete occlusions, where the available visual data are strongly reduced or inexistent (the object being no longer visible in the scene), this procedure makes it possible to make a “reasonable” guess of the object’s position in the frame where the occlusion is occurring. However, if the visual information associated to this position is further used in the tracking process, it is highly probable to be confronted to a drift phenomenon, where the occluding object takes the place of the occluded one. To avoid such situations, the only solution is to construct and use a visual appearance model of the object that can be exploited to recover, in successive frames, the missing visual information. In a certain sense, such a model defines an evolving visual memory of the object of interest.

In the following section, we construct an object appearance model that is adaptively updated in time based on the object shape and pose variation.

#### 3.2.3. Object Appearance Model

We argue that a system combining both motion and visual cues is significantly more robust and accurate than a regular tracker based solely on motion patterns. The key principle developed consists of alternating between tracking using motion information and predicting the object location in time based on visual similarity.

The object appearance model construction and update is the most challenging component of a tracking system. First, we need to decide if it is necessary to build a model for both the target object and the background or if it is sufficient to design a model solely for the object of interest. In our case, due to the real-time constraint imposed by the considered VI assistive application, we decided to develop a model only for the target. Then, a major concern refers to the reliable extraction of representative object instances necessary to update the object model. Most often, existing state of the art trackers use a single object instance, selected from the first frame (i.e., the reference image), in order to construct an object appearance model. However, such an approach quickly shows its limitations when confronted to the high dynamics of a regular urban outdoor environment. Other methods [14] propose continuously updating the appearance model with novel instances and usually formulate the task as an online learning framework. In this case, a generative/discriminative model is incrementally updated during the tracking process. However, if the object’s model is frequently updated the errors obtained during the tracking process are likely to accumulate and the system can drift away from the target.

In our case, we propose a novel, simple, yet effective strategy, which makes it possible to update the object appearance model only when such an action is required. In contrast with other tracking methods, such as [11,13] that propose to train online a CNN with object appearances observed from the past, in our case, because of the real time constraints considered, no learning process can be allowed in the online stage. Instead, the object appearance model is developed and updated based on visual similarity constraints.

More formally, the visual appearance model is defined at each frame *t* as a set $O^{t}=\left\{O_{i}^{t}\right\}$ of reference bounding boxes, determined during the tracking process from the previous frames. Let us note that the number of bounding boxes included in the appearance model can vary in time. However, for computational reasons, we consider a maximum number of five elements.

The first element included in the object appearance model set is by default the object identified by the detection module (cf. Section 3.1). For the current frame *t*, the system takes as input the eventually refined object bounding box *B^t^* determined at the end of the occlusion detection/handling module (cf. Section 3.2.2). This bounding box is doubled in both horizontal and vertical directions in order to obtain a contextual region that further serves as search area.

The object appearance model is further used to estimate the novel location of the target in the current frame. We put in competition the initial bounding box *B^t^* with all the objects instances from the appearance model $O_{i}^{t}$, in order to investigate if one of them can be more appropriate for replacing the current object bounding box *B^t^*.

To this purpose, we have considered a multiple patch matching strategy. For each object in the model, a patch is cropped from the current frame, centered in each pixel of the search area and with a size equal to the objects’ one. Each patch is then compared with the considered object to obtain a matching score. The similarity score is computed here again with the help of the DeepCompare algorithm. The patch that provides the maximum similarity score is considered to be the best-fit with respect to the current visual model object.

Let us note that this process can potentially involve a very large number of comparisons since, for each object, the number of patches to be compared with is equal to the number of pixels in the search area. To reduce the computational burden of the patch matching process, instead of the brute force search algorithm, we adopt a hierarchical, multi-resolution matching strategy with three levels of resolution inspired from the block matching method [21] used in the MPEG-4 video compression standard for motion compensation purposes.

**Figure 4:**
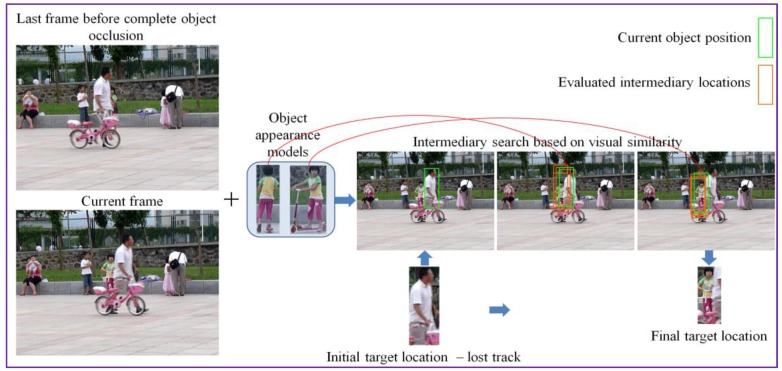
Tracker re-initialization with the correct object location using the hierarchical search algorithm.

In the case where, for a given object, the patch matching process returns a negative similarity score, we apply a final attempt to determine a fit, by enlarging (i.e., doubling the size) the search area. This can be useful in the case of long term occlusions. Then, the same recursive search process is employed. In this way, for each object in the visual appearance model a best-fit position is determined. We then select as candidate the visual object and the associated determined position that provides the maximal similarity score among all elements in the visual attention model. This process is illustrated in Figure 4.

This candidate object instance undergoes a first validation by comparing it with the initial position *B^t^*. Thus, it is considered as valid if it provides a DeepCompare similarity score superior to the one corresponding to the initial bounding box *B^t^*. In addition, we impose a so-called *temporally consistent similarity condition*. Thus, the candidate object replaces the initial bounding box *B^t^* only if its score is superior to the average score of the tracking process over the last *W* frames processed. In our work, we have set *W* = 10, which corresponds to a time interval slightly inferior to half a second. If this condition is not satisfied then the initial bounding box *B^t^* is maintained as result of the tracking algorithm at frame *t*. In a certain sense, this strategy makes it possible to enforce the information of the motion-based estimation that can be more reliable when the object suffers important deformations in shape and position.

Concerning the actualization of the visual model, an even more restrictive condition is applied. Thus, the object appearance model set is updated with a new instance whenever the visual similarity score associated to the current object in the current frame is the maximal one with respect to *all* the precedent frames within the sliding temporal window. In this case, a novel element is added to the object model set. If the number of elements in the visual model exceeds the maximum value authorized (five in our case), the oldest instance is discarded. Finally, we constrain the appearance model set not to contain target instances that have not been detected as occluded (cf. Section 3.2.2). That means that the only instances that are retained are the elements that have not undergone the shrinkage process.

In the final stage, we propose to take advantage of the target tracks in order to improve the classification module accuracy and robustness. Thus, instead of recognizing each object bounding box independently, we propose to use the previous knowledge acquired about the target as it evolves during the video sequence.

More precisely, we introduce a post-processing phase that uses the former outputs of the recognition module (cf. Section 3.1) to boost the target category in the current frame. Because, the object of interest can suffer important variation in shape and appearance, even in nearby frames, the classification result can become confuse. For this reason, we store, for each tracked object, in a *recognition vector*, the category assigned to the object during its evolution. The final object label is defined as the dominant class existent in the recognition vector.

Let us now analyze the performances of the proposed tracking approach, which has been evaluated on the challenging VOT2016 dataset.

## 4. Object Detection and Tracking: Experimental Evaluation

The proposed tracking approach is here compared in terms of accuracy and robustness with the most relevant state of the art methods.

### 4.1. The Benchmark

The proposed obstacle detection and tracking approach is evaluated on the challenging dataset considered by the Visual Object Tracking (VOT) international contest in 2016. The VOT’2016 database is composed of 60 video sequences exhibiting several challenging situations including camera motion, object occlusion, deformation, aspect ratio and size change, illumination variation, clutter or blur. All image sequences have a per-frame visual attribute labeling provided by human observers, which serves as ground truth. The VOT protocol performs two types of evaluation:

- unsupervised, where the tracking system receives as input the object bounding box from the first frame of the video sequence (the object bounding box can be provided by a human annotator or by an obstacle detection system) and then no human intervention is allowed; and
- supervised, where the tracker is locally re-initialized with the object bounding box (from the ground truth) if the target element is lost (tracking failure).

The proposed framework was implemented in Microsoft Visual C++, with OpenCV library. For the deep learning purposes, we have used the Caffe framework [18] with cuDNN optimization. The average processing time at the run-time stage is around 20fps on a computer equipped with Intel 3.4 GHz CPU, 64 GB RAM and NVIDIA GeForce 1080Ti GPU.

We compared the experimental results obtained by 14 state-of-the-art trackers: the GOTURN [15], Staple [13], C-COT [14] (the winner of the VOT 2016 challenge), ASMS [2], MAD [3], TricTRACK [4], KCF2014 [5], STC4 [6], SWCF [7], CDTT [8], DAT [9], STC [10] and TCNN [11].

For the GOTURN algorithm [15], the default parameters and the source codes provided by the authors are used in all the evaluations. For the other trackers, we have considered the results validated by the VOT2016 technical committee and reported in [12].

### 4.2. Evaluation Metrics

One of the most popular measures, extensively used in the state of the art for evaluation purposes is the so-called *center prediction error*, defined as the difference between the object center, predicted using the tracking approach, and the ground-truth data. This measure offers the advantage of simplicity, being straightforward to implement. However, as indicated in [12], the center prediction error is highly sensitive and strongly depending on the quality of the ground truth annotation. In addition, it completely ignores the size of the target object and does not reflect the target apparent failure.

To obtain an evaluation measure that can more precisely reflect the quality of the obtained bounding boxes, we have adopted the so-called *accuracy* measure, recently considered by the VOT challenge [12]. The accuracy is defined for each frame *t* as the region overlap score between the predicted target bounding box position and the ground-truth region (Equation (5)):

(3) $$\Phi ={\left\{\phi_{t}\right\}}_{t=1}^{N},\;with\;\phi_{t}=\frac{\left|R_{t}^{G}\cap R_{t}^{T}\right|}{\left|R_{t}^{G}\cup R_{t}^{T}\right|},$$

where $R_{t}^{G}$ denotes the object ground-truth region at time *t*, $R_{t}^{T}$ is the region size at time *t* indicated by the tracking system *T*, *N* is the total number of frames for the considered video sequence, |.| denotes the number of pixels of the considered regions, and ∪ and ∩, respectively, represent the union and intersection of the two regions.

The overlap can be interpreted as:

(4) $$\Phi =\frac{TP}{TP+FN+FP}\;,\;$$

where TP denotes the number of true positive pixels, FN is the number of false negative pixels and FP represents the number of false positive pixels.

The overlap measure can be globalized over the entire video sequence by taking the average value over all the video frames [12]:

(5) $$\bar{\Phi }=\frac{1}{N}\overset{N}{\underset{i=1}{\sum }}\phi_{t},\;$$

Another measure that is extensively used [12] for evaluating a tracker performance is the *failure rate*. The failure rate considers the evaluation problem in a supervised manner and involves a human observer which reinitializes the tracker at each failure. The average number of manual interventions per 30 frames is recorded and used as a comparative score.

To be able to evaluate separately the influence of the various conditions that can appear in the videos (e.g., camera motion, illumination changes, object occlusion, size change, target motion change), we have adopted the globalization protocol considered by the VOT’2016 challenge. Here, the videos of the database are split into smaller videos, with a size of *N*_s_ frames, corresponding to each of the considered situations. The *N*_s_ parameter takes values within a [*N*_li_, *N*_hi_] interval that is automatically generated by the VOT’2016 toolkit.

By averaging the average overlap measure $\bar{\Phi }$ on a set of sequences with *N*_s_ frames, we obtain the expected average overlap ${\hat{\Phi }}_{N_{s}}$. Finally, expected average overlap score (EOS) is defined as described by the following equation:

(6) $$\hat{\Phi }=\frac{1}{N_{\mathrm{hi}}-N_{\mathrm{li}}}\underset{N_{s}=N_{\mathrm{li}}:N_{\mathrm{hi}}}{\sum }{\hat{\Phi }}_{N_{s}},\;$$

### 4.3. Quantitative Evaluation

**Figure 5:**
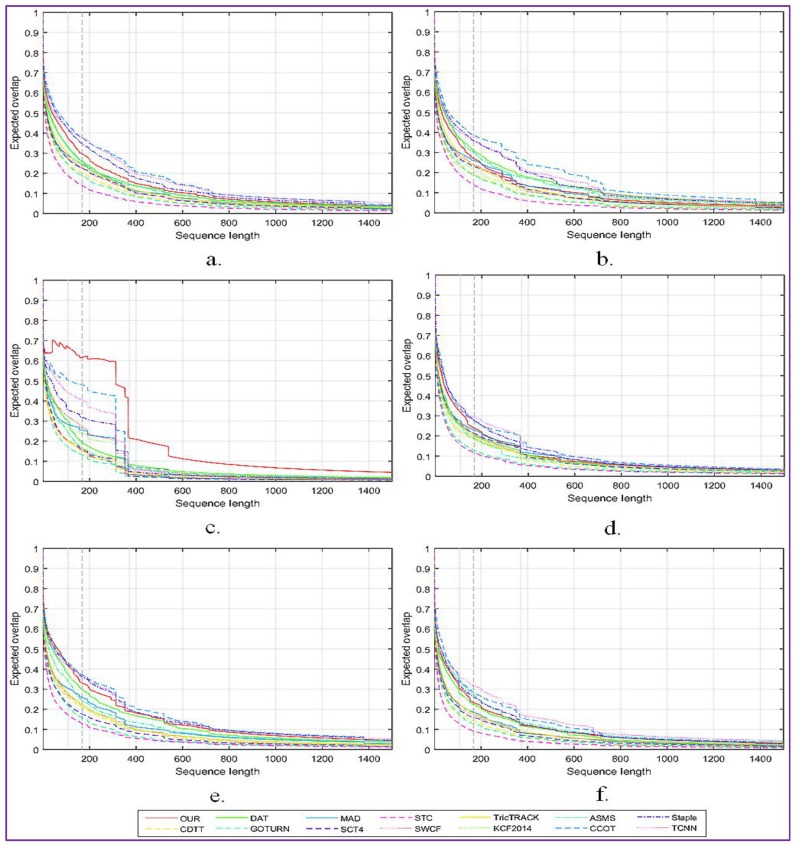
The expected average overlap score (EOS) for the proposed tracking approach and comparison with state of the art methods: (**a**) The overall EOS scores. The EOS scores in videos with important: (**b**) camera motion; (**c**) illumination changes; (**d**) object occlusion; (**e**) object size change; and (**f**) target motion change.

The obtained results are generated using the toolkit set up by offered by the VOT committee. The overall expected overlap scores for the proposed tracker and for 13 other relevant tracking systems, denoted C-COT [14], Stapler [13], ASMS [2], MAD [3], TricTRACK [4], KCF2014 [5], SCT4 [6], GOTURN [15], SWCF [7], CDTT [8], DAT [9], STC [10] and TCNN [11], are presented in Figure 5a.

In addition, Figure 5 presents a more detailed analysis of the impact of the following factors: camera motion (Figure 5b), illumination changes (Figure 5c), object occlusion (Figure 5d), object size change (Figure 5e) and target motion change (Figure 5f).

As can be observed, the proposed approach shows the highest robustness when important variations of the light intensity are presented on the scene. When the target is occluded or has an important motion, we obtain equivalent performances with the C-COT method. However, the proposed tracking procedure is more sensitive to important camera motion than state of the art systems such as Staple, C-COT or TCNN.

**Figure 6:**
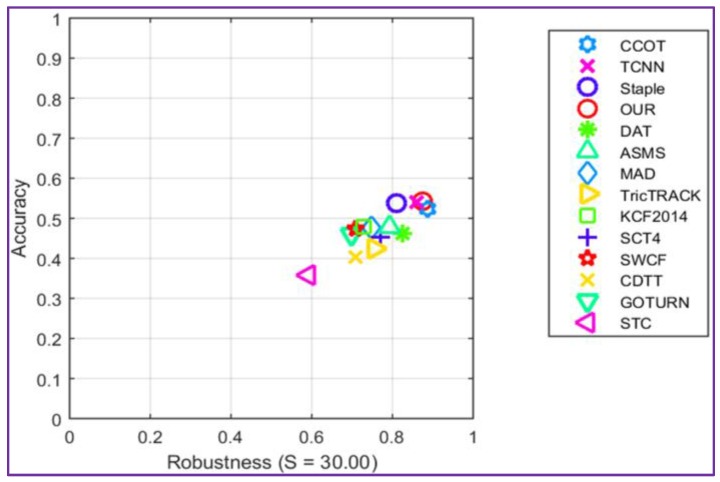
Comparison with state of the art on the VOT 2016dataset using the robustness-accuracy plot.

The accuracy-robustness score plot is presented in Figure 6. It can be observed that the proposed tracker shows comparable performances in terms of accuracy and robustness when compared with the winner C-COT of VOT2016 challenge.

The major improvement of the proposed method is the processing speed, which is about four times faster than the analyzed trackers that return similar performances scores. This result is explained by the fact that our tracker avoids any on-line learning stage, in contrast with C-COT, where the object appearance model is learned online by a CNN. On the opposite side, when compared with the GOTURN algorithm that also uses only offline trained convolutional networks and works in real-time, our method shows a significant improvement in accuracy (more than 10%). In addition, in terms of the number of failures, our approach shows an improvement with more than 75%.

Table 1 reports the average accuracy and the number of failures for each tracker.

From the experimental results presented in Table 1, it can be observed that our tracker has the highest accuracy of 0.54, occupies the fourth rank in terms of failure rate, while offering a processing speed that is four time faster than its top competitors.

To provide a qualitative insight of the behavior of the proposed tracking approach, the following section presents the results obtained on some challenging video sequences.

### 4.4. Qualitative Evaluation

**Figure 7:**
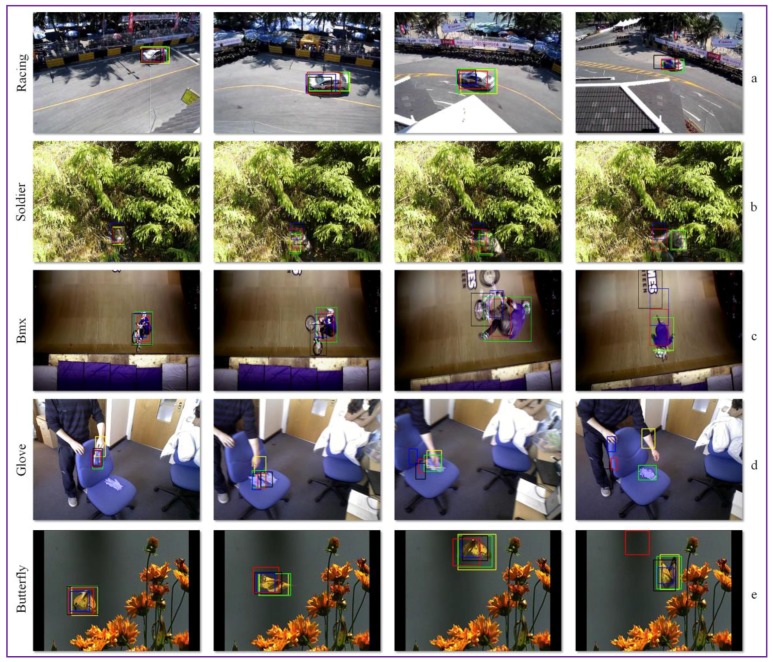
Experimental tracking results on: (**a**) Racing; (**b**) Soldier; (**c**) Bmx; (**d**) Glove; and (**e**) Butterfly videos. Green: Our results; Blue: C-COT [14]; Yellow: GOTURN [15]; Red: Staple [13]; Black: TCNN [11].

In Figure 7, we present the tracking results obtained by the proposed approach, compared to the top three state of the art trackers, Staple, C-COT and TCNN. We selected from the VOT 2016 corpus five challenging video sequences, denoted as Racing, Soldier, Bmx, Glove and Butterfly.

In the Racing and Butterfly image sequences, all trackers are able to follow the object of interest. However, because of the relatively important change in object appearance, the state of the art trackers (i.e., C-COT, Staple or TCNN) cannot adaptively modify the object bounding box in order to capture its entire shape. Thanks to the continuous update of the object appearance model and to the intelligent adjustment of the object’s bounding box, our method is able to perform more accurately.

For the Bmx video, we can observe that all the state of the art trackers tend to drift because the model is not correctly updated in time. In the Glove sequence the object of interest suffers an important change in shape and undergoes an unnatural motion. In addition, two visually similar objects are present in its vicinity (i.e., the users’ hands). Because all state of the art CNN trackers learn on-line the object features, when a new, visually similar element enters the scene, the tracker tends to shift to the novel objects.

In the case of the Soldier video sequence, the state of the art trackers (i.e., C-COT, Staple or TCNN) are updated with sub-optimal object instances and tend to drift in time. For our method, due to the occlusion identification and handling stage, this behavior rarely occurs.

Let us now describe the application considered in this paper: the ***DEEP-SEE*** visually impaired navigational assistant is detailed in the following section.

## 5. The *DEEP-SEE* Navigational Assistant

Recent statistics, relative to people with visual disabilities, published by the World Health Organization (WHO) [22] in August 2014, show that more than 0.5% of the total population suffers from visual impairments (VI). Among these, 39 million are completely blind. Unfortunately, by the year 2020, worldwide the number of individuals with VI is estimated to double [23].

The visually impaired people are facing numerous challenges when performing daily activities (e.g., navigation in indoor/outdoor scenes, people recognition, safe travelling or independent shopping) which can severely impact the quality of their private and professional life. Even though modern technology has found its way into most aspects of people life, in the context of VI, the traditional white cane or the trained walking dog remains the most common mobility aids [24].

Within this context, the development of an autonomous assistive device designed to facilitate the user cognition over the environment and to increase the VI autonomy and safety when navigating in novel places is a crucial challenge.

In the state of the art, various electronic travel aids based dedicated to people with visual disabilities were introduced based on either sensor networks [25,26,27,28,29,30] or computer vision techniques [30,31,32,33,34,35]. The assistive devices based on sensorial substitution of human vision can be very effective when used in indoor environments or when employed to detect large, flat structures. However, in the case of outdoor urban scenarios the systems sensitivity becomes prohibitively high. The computer vision systems prove to be highly effective in identifying fast moving objects in outdoor scenes. However, the systems become sensitive in some particular conditions, notably when: (a) the video camera exhibits sudden movements; (b) the video stream is blurred; and (c) the object moves on a parallel path with the VI user. The state of the art analysis highlights the following conclusions: (1) no method can accomplish in a satisfactory degree all the features that are required for being accepted by the VI community, i.e., work in real-time, function in both indoor and outdoor scenarios, present high robustness and accuracy scores, be portable and user-friendly; and (2) every system has its own advantages and limitations over the others but no method is accurate and robust enough to completely replace the white cane.

In this section, we introduce a novel assistive device, which exploits the tracking methodology introduced in Section 3 to detect, track and recognize in real-time both static and dynamic obstacles situated in the vicinity of a VI user when navigating in outdoor environments.

### 5.1. System Architecture

When designing the proposed architecture the following set of requirements were considered: *processing speed* (the system should function in real-time and to alert immediately the user about an obstacle situated at a distance inferior to 1.5 m), *robustness* (the system should not by influenced about the scene dynamics or lighting conditions), *coverage distance* (the maximum distance between the user and an object for which detection can be performed), *object type* (the system should be able to detect any type of object regardless on its position, shape, size or if the object is static or dynamic), *portability* (the system should be light, ergonomic and easy to wear), and *friendliness* (the system should be easy to learn without any training).

**Figure 8:**
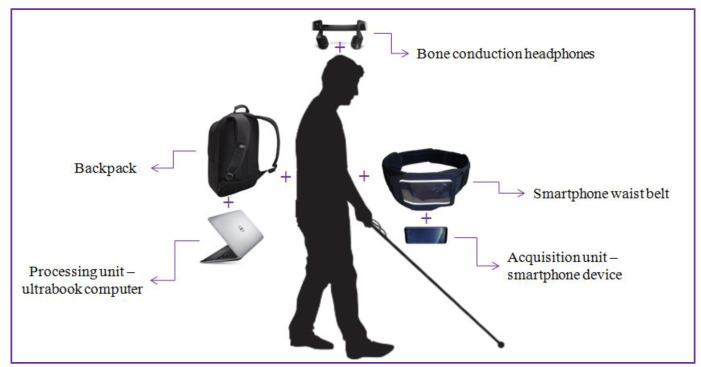
The ***DEEP-SEE*** hardware components.

Figure 8 illustrates the hardware configuration proposed. The ***DEEP-SEE*** system is composed of a regular smartphone device (Samsung S8+, Samsung Electronics Co., Suwon, Korea) functioning as a video acquisition device, Bluetooth bone conducting headphones (AfterShokz Bluez 2 Voxlink LLC, East SyracuseOnondaga, New York, NY, USA) used to transmit the acoustic warning messages to the VI user, an ultrabook computer (Dell XPS running on an Nvidia GTX 1050 GPU, Nvidia Corporation, Santa Clara, CA, USA) used as backpack processing unit, a smartphone waist belt and a backpack. The total weight of our system is inferior to 2 kg.

**Figure 9:**
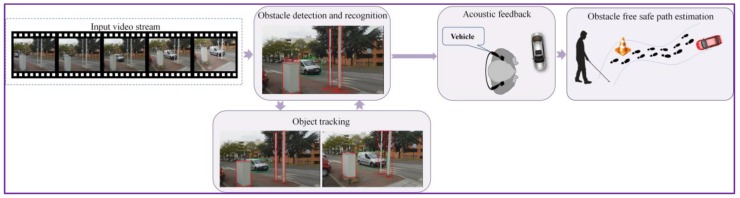
The ***DEEP-SEE*** software architecture.

At the software level, the ***DEEP-SEE*** architecture adopts a modular framework, composed of the following three units: object detection and recognition (cf. Section 3.1), object tracking (cf. Section 3.2) and acoustic feedback (Figure 9).

Finally, regarding the user interaction aspects, ***DEEP-SEE*** provides an intuitive and limited feedback by using verbal warning messages. We introduce an acoustic warning protocol able to prioritize, based on the degree of danger of the recognized obstacles, the acoustic alerts and transmit a message only when such an action is required (i.e., in order to avoid collision).

#### 5.1.1. Acoustic Feedback

The acoustic feedback is designed to improve cognition of the visually impaired people over the outdoor environment by transmitting warning messages regarding the recognized obstacles (either static or dynamic) situated in his/her near surrounding. The major constraint that needs to be taken into account is to transmit warning messages fast enough, so that the VI user can walk normally while avoiding dangerous situations. We decided to use bone conduction headphones, in order to satisfy the hands free condition imposed by the VI people and enable the user to keep hearing other external sounds from the surroundings.

Most assistive devices dedicated to VI people existent on the market [28] try to exploit the sense of touch as a natural substitution to the visual sense. In this case, tactile displays (that involve arrays of vibrators as skin indentation mechanisms for fingers or palms) are used in order to transmit warning messages. However, after consulting VI users from several blind associations, we decided to use acoustic warnings rather than tactile stimulation. Most of the VI users consider the tactile display invasive because they require an actual physical contact with the person’s skin. In addition, the information acquired from the haptic sense is insufficient to capture the overall semantics of the scene.

In the context of ***DEEP-SEE***, the semantic interpretation of the recognized objects and the effective transmission of information to VI people are the key elements. The acoustic feedback module is designed to be intuitive and does not require extensive and laborious training phases to understand the technical functionalities.

The key principle that has been adopted respects the VI user recommendations and fulfills the following two rules: (1) the user should not be overwhelmed with too many and useless alerts; and (2) the system should minimize all interference with other senses such as auditory and touch.

We decided to use verbalized messages rather than a beeping strategy (with sound patterns transmitted on different frequencies) so that the user is informed about the presence of an approaching obstacle, its type or degree of danger, time to collision and the physical position of the detected obstruction.

#### 5.1.2. Estimation of the Degree of Dangerousness and Prioritization of Messages

**Figure 10:**
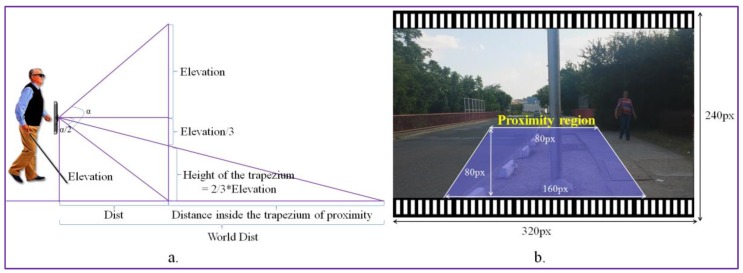
Obstacle classification as urgent and normal based on their position relative to the VI user: (**a**) object distance estimation; and (**b**) the trapezium of interest size expressed in pixels.

For each detected obstacle, we determine its degree of danger by estimating the object’s position relatively to the video camera. We propose to use a trapezium of interest defined in the space of the video frame in order to define the user’s proximity area. The dimensions of the trapezium (in pixels) are presented in Figure 10. The acquired videos are re-sized at a size of (320 × 240 pixels), which corresponds to the second lower resolution supported by the camera. We use as an acquisition device the video camera embedded on a smartphone with an angle of view α = 69 degrees (value provided by the manufacturer). We set the trapezium height at 1/3 of the video frame height. Nevertheless, the size of the trapezium can be adjusted in a pre-calibration step by the user.

The smartphone is attached to the VI using a waist belt at an average elevation (*E*) of 0.8–1 m (which corresponds to the height of the waist of a medium person between 1.60 and 1.80 m height). Then, we estimate the distance *Dist* between the user and the first visible area on the floor as:

(7) $$Dist=\frac{E}{tg\left(\alpha /2\right)};$$

In the image space, the visible area defined by the *Dist* value corresponds to the pixels located at the bottom of the trapezium of interest.

The principle that is adopted consists of using the trapezium of interest as an *alert area* and to launch warning messages only for objects whose position in the image intersect this trapezium.

We can estimate the distance in meters (in the real world) between the user and the obstacles detected in the alert area (Figure 10) as described by the following equation:

(8) $$WorldDist=Dist+\frac{2\cdot E/3}{\frac{E}{3}\cdot tg\left(\alpha /2\right)}=3Dist\approx 6\;m;$$

The *WorldDist* value corresponds to the distance to the user of an object that is entering in the alert area. In our setting, with the above-considered parameters, the *WorldDist* value is of approximately 6 m. We consider this value as a reasonable choice to decide that an object becomes too close to the user and needs to be notified as urgent (*U*). Thus, an object obstacle is marked as urgent if it is situated in the proximity of the blind/visual impaired person inside of the trapezium of interest. For objects situated within the trapezium of interest we estimate an average collision time of 1–2 s. Otherwise, if it is located outside the trapezium, the obstacle is categorized as non-urgent or normal (N). By employing the area of proximity, we can prevent the system to launch acoustic warning messages for all the detected objects existent in the scene. We adopted this strategy in order not to overwhelm the user with too much information. However, within the trapezium of interest multiple static/dynamic obstacles can be encountered. The recognized objects are further analyzed in order to differentiate between the various elements and to determine their degree of danger. To this purpose, we exploit the object recognition process described in Section 3, which makes it possible to identify the five main categories dedicated to the VI-navigation purposes: vehicle, motorcycle, bicycle, pedestrian and obstruction.

Table 2 presents the set of acoustic warnings proposed in descending order of priority (i.e., 1 represents the element with the highest importance).

As it can be observed from Table 2, the dynamic obstacles with a potential high degree of dangerosity (e.g., vehicle or motorcycle) situated in the non-urgent area will receive a priority score of 4 and 5, respectively, which is superior to relevance scores of pedestrians or static obstacles (6 and 7) located within the urgent area. When a vehicle or motorcycle *approaches* the VI user, even if not located within the trapezium of interest, the system will generate an acoustic warning and will inform the VI user about its presence instead of the existence of a static obstacle or a pedestrian near him/her. Moreover, static obstacles or pedestrians that are not located in the alert area do not generate any warning.

**Figure 11:**
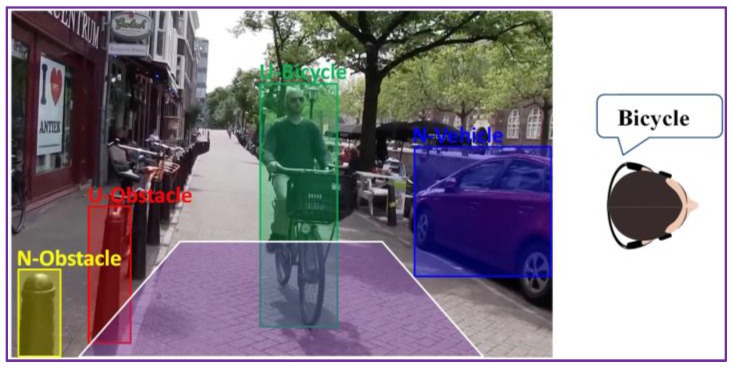
Acoustic warning message prioritization based on the object semantic interpretation.

This strategy is illustrated in Figure 11, where multiple objects are present in the scene. The system will assign a relevance score to each element and then transmit a warning message only for the most important object, which is in this case “Urgent bicycle”.

Finally, to provide information regarding the relative position of the detected object, the acoustic warning messages are encoded in stereo using either right, left or both channels simultaneously. Thus, when the obstacle is situated on the left (right) side of the subject the message is transmitted on the left (right) channel of the bone conducting headphones. For frontal objects, both channels are used simultaneously to transmit the warning. In addition, to avoid confusing the VI user with too much information, the warning messages are sent with a frequency rate inferior to two seconds, regardless of the scene dynamics.

### 5.2. VI Navigational Assistance: Experimental Evaluation

The *overall **DEEP-SEE*** navigational assistant has been tested on multiple, complex outdoor environments by using the video dataset of [16] that was recorded with the help of visually impaired people. The dataset contains 30 video sequences with an average duration of 10 min, acquired at 30 fps and at a resolution of 320 × 240 pixels.

**Figure 12:**
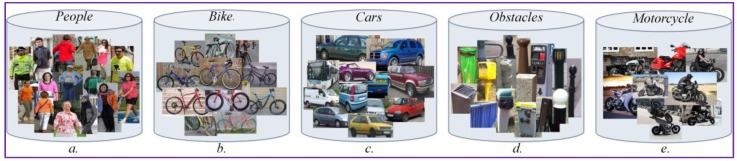
Examples of object instances included in the testing database. (**a**) People instances; (**b**) Bycicles instances; (**c**) Vehicles instances; (**d**) Static obstacles; (**e**) Motocycles instances.

Because the dataset was recorded with the help of visually impaired people in real urban scenes and not in simulated environments, the videos are trembled, cluttered and include important camera and background movement. In addition, various types of dynamic and static objects are present. In Figure 12, we give some examples of static/dynamic objects existent in the video dataset that have been used for testing. As it can be observed, we have selected the following five major categories: vehicles, motorcycles; bicycles, pedestrians and static obstacles. The considered object classes were chosen according to the most often encountered obstacles in outdoor navigation. All the static objects, such as pillar, fences, traffic signs, stairs, trees, and benches, encountered by the VI user during the outdoor navigation were included in the static obstruction class. We adopted this strategy in order to be consistent with the evaluation provided in [16].

Using the ground truth data, the performance of the obstacle detection and classification modules are evaluated using traditional objective parameters such as Precision (*P*), Recall (*R*) and *F*1 score (*F*1), defined as:

(9) $$P=\frac{TP}{TP+FP},\;R=\frac{TP}{TP+FN},\;F1=\frac{2\cdot P\cdot R}{P+R};$$

where TP is the number of true positive elements (correctly detected and classified objects), FP is the number of false positive (incorrect detected/classified objects) and FN false negative elements (missed detected/classified objects). For the evaluation, we used the video dataset of [16] where the ground truth elements represent the number of objects, for each category, that need to be detected and classified. Table 3 summarizes the experimental results obtained.

We have compared the proposed approach with our previous method introduced in [16], which according to the recent state of the art review presented in [36], offers some of the highest recognition performances.

The obtained results highlight that the average precision and recall scores are around 90% which means that the proposed framework is able to detect and classify, with high accuracy, both dynamic and static obstacles. When compared with our previous method in [16], our system achieves an improvement with more than 5% of the F1-score, which shows the interest of exploiting CNN-based approaches for detection and recognition tasks.

In terms of computational speed, when implementing the whole framework on a regular ultrabook computer, running on an Nvidia GTX 1050 GPU, the average processing speed is around 20 fps.

**Figure 13:**
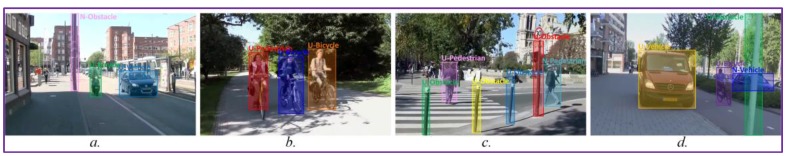
Experimental results on the video dataset acquired by VI users. (**a**) Evry1 video sequence; (**b**) Park image sequence; (**c**) Paris2 video sequence; (**d**) Evry2 image sequence.

In Figure 13, we present some results obtained. The category of each detected obstacle is also presented.

From the experimental results presented in Figure 13, it can be observed that the ***DEEP-SEE*** prototype is able to correctly detect, track and recognize various object encountered by the VI user during the outdoor navigation. In addition, the system proves to be robust to the scene dynamics or to various changes in the light intensity or object appearance.

## 6. Conclusions and Perspectives

In this paper, we have introduced a novel navigational assistant prototype designed to increase the mobility and safety of visually impaired people when navigating in outdoor environments. The proposed framework, denoted ***DEEP-SEE***, jointly exploits computer vision algorithms and deep convolutional neural networks and is designed to detect, track and recognize, in real time, both static and dynamic obstacles encountered during navigation in crowded scenes. The system does not require any initial a priori knowledge about the object size, shape or initial position. The semantic interpretation of the recognized objects and the effective transmission of information to a VI people are the key elements. The degree of danger of the detected objects is also evaluated by estimating the object’s relative position with respect to the to the VI user. The user feedback is transmitted as a set of acoustic warnings, through bone conducting headphones.

From a methodological point of view, the core of the approach relies on a novel object tracking method that uses two CNNs for taking into account both motion patterns and visual object appearances. We evaluated the proposed system on two video datasets: the Visual Object Tracking 2016 database composed of 60 videos. The experimental results obtained on the challenging VOT 2016 datasets demonstrate that the proposed detection and tracking system is capable to obtain state of the art performances by alternating between two CNNs, respectively, trained with motion and visual patterns. In terms of accuracy and robustness, the tracking system returns similar performances with the winner of the VOT2016 challenge while being four times faster.

Concerning the evaluation of the ***DEEP-SEE*** prototype for VI assistance purposes, we have considered the video dataset of [16] recorded with the help of VI users. The proposed methodology led to a gain of 5% in terms of F1 score when compared with [16].

For further work and developments, we propose to integrate the proposed framework in a complete platform that allows additional functionalities, such as crossings detector, face recognition, shopping assistance, and guiding system, that may help a VI user to reach a desired destination. Next, we propose extending the proximity region and define two areas of interest, both with a trapezoidal shape, in the near surrounding of a user: one situated on the walking path and the other at the head level. Using this strategy, we can launch acoustic warning messages for obstacles situated at the head level, such as tree branches, hanging signs or other dynamic flying objects, and not solely for object located on the ground.

In addition, we envisage testing the system in real-life scenarios with actual visually impaired users. Finally, let us observe that elderly VI users may also present hearing disabilities. In this case, an alternative, haptic encoding scheme should be envisaged instead of the acoustic warning approach.

## Figures and Tables

**Table 1:** Experimental results obtained on the VOT 2016 dataset.

Tracker	Year	Where	Accuracy	Failure Rate
Our	-	-	**0.54**	1.17
GOTURN	2016	ECCV	0.42	2.46
Staple	2016	CVPR	**0.54**	1.15
C-COT	2016	ECCV	0.52	**0.85**
TCNN	2016	CVPR	0.53	0.96
DAT	2014	CVPR	0.46	1.72
ASMS	2015	PRL	0.48	1.87
MAD	2016	SPIE	0.48	1.81
TricTRACK	2015	ICCV	0.43	2.08
STC	2016	ECCV	0.36	3.61
KCF2014	2015	PAMI	0.48	2.03
STC4	2016	CVPR	0.45	1.95
SWCF	2016	ICIP	0.47	2.37
CDTT	2015	CVPR	0.41	2.08

**Table 2:** Warning messages generated by the ***DEEP-SEE*** system.

Order of Relevance	Recognized Object	Acoustic Warning
1	Vehicle	Urgent vehicle
2	Motorcycle	Urgent motorcycle
3	Bicycle	Urgent bicycle
4	Vehicle	Normal vehicle
5	Motorcycle	Normal motorcycle
6	Pedestrian	Urgent pedestrian
7	Obstruction	Urgent static obstacle
8	Bicycle	Normal bicycle

**Table 3:** Experimental results obtained on the dataset acquired using VI people.

Obstacle Type	Ground Truth	Precision		Recall		F1-Score	
		*DEEP-SEE*	[16]	*DEEP-SEE*	[16]	*DEEP-SEE*	[16]
Vehicle	431	0.94	0.94	0.92	0.92	0.93	0.92
Bicycle	120	0.91	0.87	0.90	0.69	0.90	0.77
Pedestrian	374	0.95	0.89	0.95	0.91	0.95	0.90
Static obstruction	478	0.90	0.90	0.87	0.79	0.88	0.84
TOTAL	1403	0.92	0.90	0.91	0.83	0.91	0.86
